# Retraction: The mediating role of math self-efficacy between school-based parental involvement and math performance among students in Southeast Asia: evidence from PISA 2022

**DOI:** 10.3389/fpsyg.2026.1930576

**Published:** 2026-07-13

**Authors:** Frontiers Editorial Office

The journal retracts the 29 August 2025 article cited above.

Following publication, concerns were raised regarding data misrepresentation. Specifically, it was identified that the reported items did not correspond to the original items from the Programme for International Student Assessment (PISA).

During the subsequent investigation, conducted in accordance with the journal's policies, the authors did not provide a satisfactory explanation for these discrepancies. As a result, the editors no longer have confidence in the validity and reliability of the findings presented in the article.

This retraction was approved by the Chief Editors of Frontiers in Psychology and the Chief Executive Editor of Frontiers.

Frontiers would like to thank the concerned reader who contacted us regarding the published article.

